# Spigelian Hernia Masquerading as Chronic Lower Abdominal Pain: A Case Report

**DOI:** 10.7759/cureus.83098

**Published:** 2025-04-27

**Authors:** Vijaykharthik LK, Ganesh Guru K, T Raghupathy

**Affiliations:** 1 General Surgery, Sree Balaji Medical College and Hospital, Chennai, IND

**Keywords:** abdominal wall hernia, chronic abdominal pain, ct diagnosis, open mesh repair, rare hernia, spigelian hernia, surgical management

## Abstract

Spigelian hernias are rare and often overlooked causes of lower abdominal pain due to their deep location and subtle clinical signs. This case involves a 55-year-old woman with a five-year history of intermittent right lower abdominal discomfort that gradually worsened with physical activity. Clinical examination was inconclusive, prompting further evaluation with imaging, which revealed a defect in the Spigelian fascia with bowel herniation. She underwent successful open mesh repair, with an uneventful recovery and complete resolution of symptoms. This case highlights the diagnostic challenges associated with Spigelian hernias and underscores the importance of maintaining a high index of suspicion in patients with persistent, unexplained abdominal pain, as early imaging and surgical intervention can lead to excellent outcomes.

## Introduction

Spigelian hernia, also known as lateral ventral hernia, is a type of abdominal wall hernia that occurs through a defect in the Spigelian fascia-the aponeurotic layer between the rectus abdominis muscle medially and the semilunar line laterally. It accounts for only 0.1%-2% of all abdominal wall hernias, making it an uncommon clinical entity. Due to its interparietal location and often subtle or nonspecific symptoms, Spigelian hernia poses a significant diagnostic challenge, particularly in obese patients where a palpable mass may be absent [1].

Risk factors for Spigelian hernia include conditions that increase intra-abdominal pressure, such as chronic cough, constipation, heavy lifting, obesity, and prior abdominal surgery. While some patients remain asymptomatic, others may present with localized pain, swelling, or discomfort-symptoms that may be chronic and misattributed to other gastrointestinal or musculoskeletal causes [2].

Prompt diagnosis is crucial, as these hernias are prone to incarceration and strangulation due to their typically narrow neck. Imaging, especially computed tomography (CT), plays a vital role in confirming the diagnosis. Surgical repair, either open or laparoscopic, remains the definitive treatment [3].

This report presents a case of Spigelian hernia in a middle-aged woman with chronic lower abdominal discomfort, highlighting the importance of high clinical suspicion, the role of imaging in diagnosis, and the effectiveness of surgical intervention.

## Case presentation

A 55-year-old woman with a BMI of 32 presented to the surgical outpatient department with complaints of intermittent pain and localized swelling in the right lower quadrant of the abdomen for the past five years. The discomfort was dull-aching in nature and aggravated by physical activity, particularly during prolonged standing or lifting. There was no history of trauma, previous abdominal surgery, weight loss, vomiting, or changes in bowel habits.

**Figure 1:**
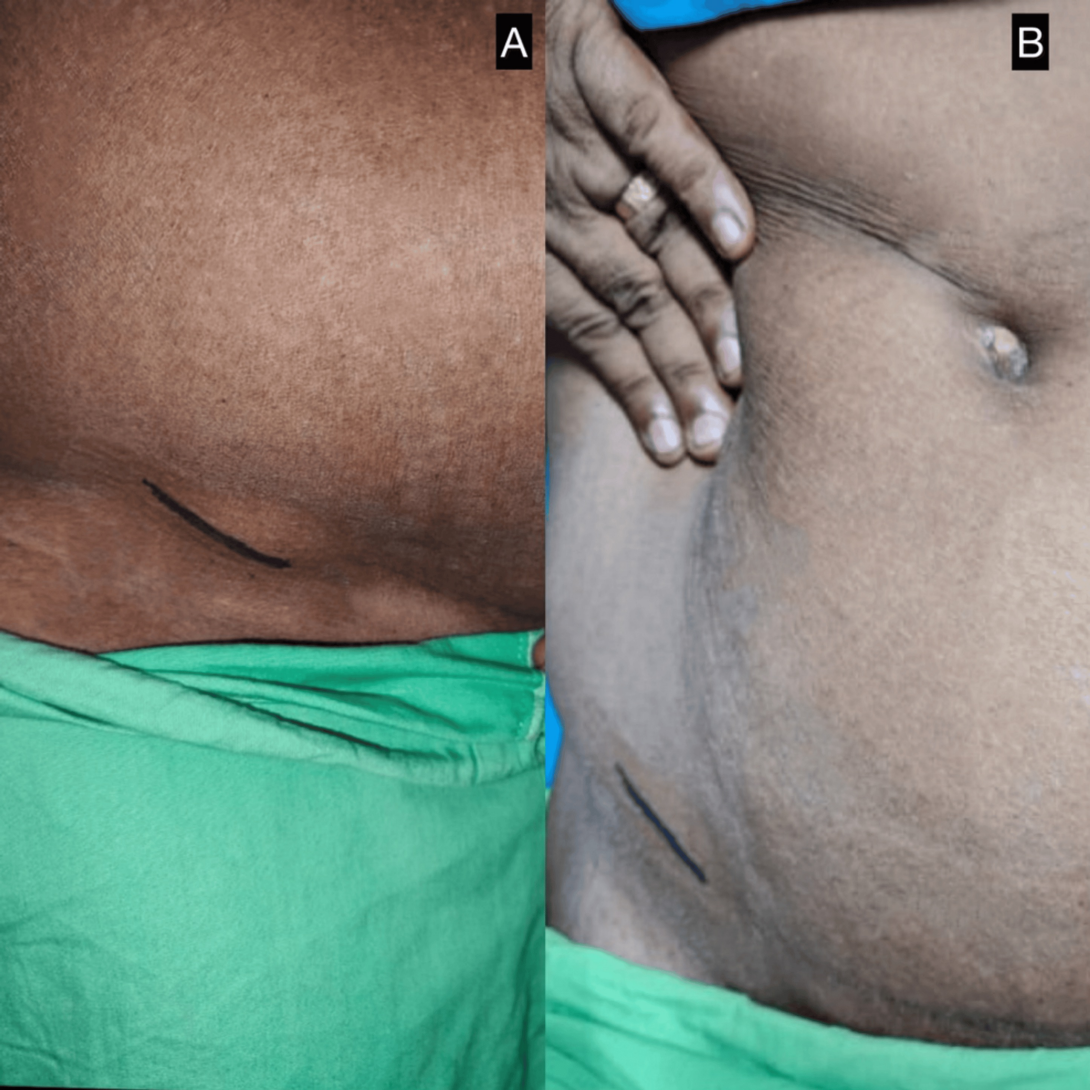
Preoperative clinical photograph of the Spigelian hernia (A) Lateral view showing localized bulge in the right lower quadrant suggestive of Spigelian hernia. (B) Anterolateral view of the same patient highlighting the hernia location marked over the Spigelian belt.

On clinical examination, the patient had localized tenderness in the right lower abdomen; however, a localized visible bulge was evident on inspection, and no palpable mass was felt on palpation (Figures 1A, 1B). Given the chronicity of symptoms and the absence of clear clinical findings, further radiological evaluation was pursued.

**Figure 2:**
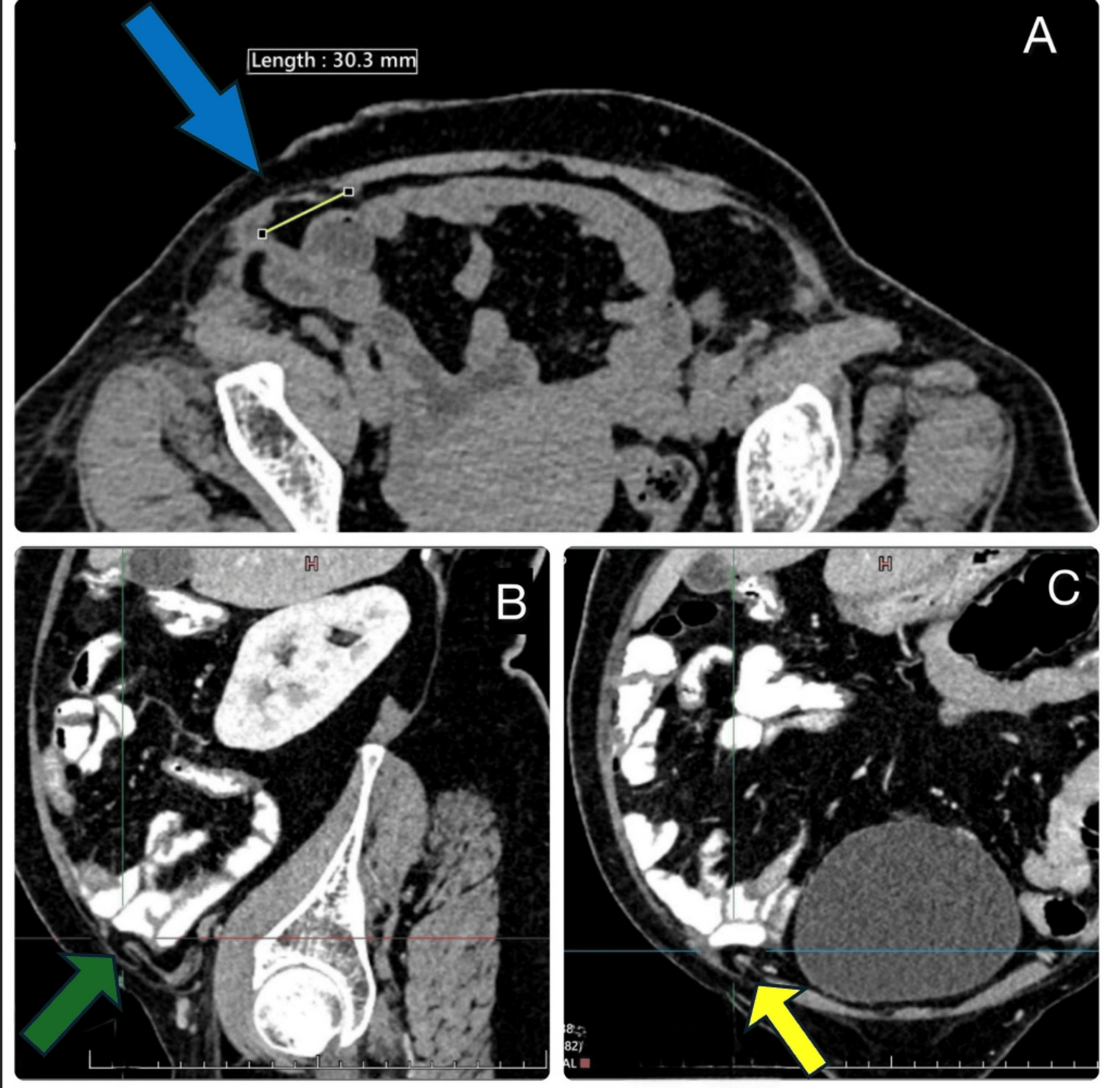
CECT abdomen revealed a defect in the Spigelian fascia on the right side, with herniation of bowel loops consistent with a Spigelian hernia (A) Axial CECT of the abdomen revealed a defect in the Spigelian fascia on the right side (blue arrow), with herniation of bowel loops consistent with a Spigelian hernia. There was no evidence of bowel obstruction, incarceration, or strangulation, (B) sagittal CECT abdomen shows a defect as mentioned above (green arrow), and (C) coronal CECT abdomen shows a defect as mentioned above (yellow arrow). CECT: contrast-enhanced computed tomography

A contrast-enhanced CT (CECT) scan of the abdomen revealed a well-defined defect in the Spigelian fascia on the right side, with herniation of bowel loops consistent with a Spigelian hernia. There was no evidence of bowel obstruction, incarceration, or strangulation (Figures 2A-2C).

**Figure 3:**
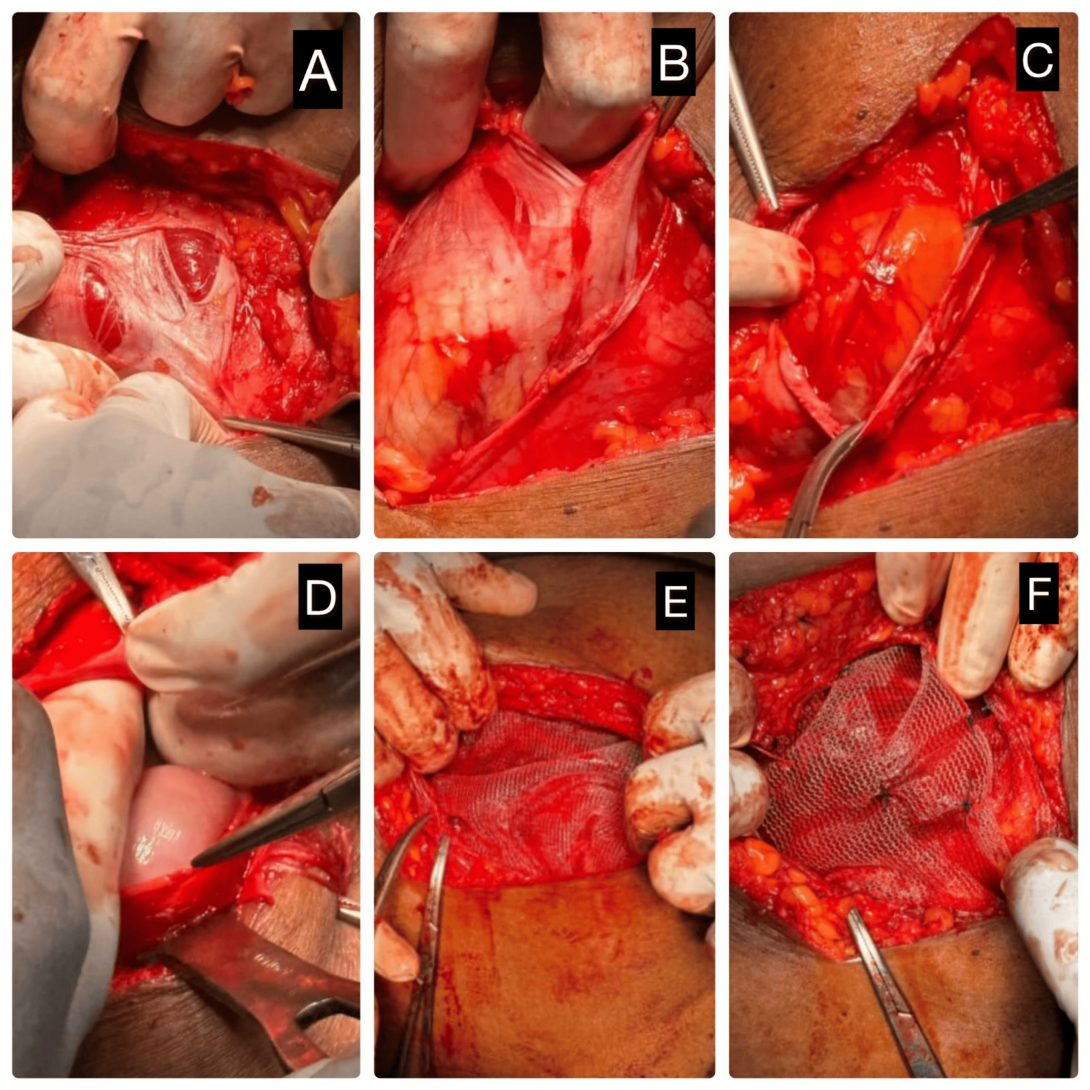
Intraoperative steps of open mesh repair for Spigelian hernia (A-F) Sequential intraoperative images demonstrating (A) identification of the hernial sac through the defect in the Spigelian fascia, (B) dissection and mobilization of the hernial sac, (C) exposure of hernia contents, (D) reduction of hernia contents into the peritoneal cavity, (E) placement of synthetic mesh over the defect, and (F) mesh fixation completing the repair.

The patient was taken up for elective surgical repair. Under general anesthesia, an open approach was adopted. Although laparoscopic repair is an established option for Spigelian hernia, an open approach was preferred in view of the chronic nature of the hernia, anticipated adhesions, and the patient's obesity, which could complicate laparoscopic access and increase operative risks. Intraoperatively, a fascial defect was identified in the Spigelian zone, and herniated bowel loops were visualized without signs of ischemia or strangulation. The herniated contents were reduced, and the defect was reinforced using onlay polypropylene meshplasty (Figures 3A-3F).

Postoperative recovery was uneventful. The patient was mobilized on the first postoperative day and discharged on the third postoperative day with advice to avoid strenuous activities for a few weeks. On follow-up visits at two weeks and three months, the patient remained asymptomatic, with no signs of recurrence or postoperative complications.

## Discussion

Spigelian hernia is a rare and frequently overlooked type of abdominal wall hernia that occurs through a defect in the Spigelian fascia, typically found along the lateral border of the rectus abdominis muscle. Representing less than 2% of all abdominal wall hernias, its interparietal location and subtle presentation often result in nonspecific symptoms, making it challenging to diagnose clinically [4].

Patients often report intermittent pain or discomfort in the lower abdomen, sometimes accompanied by a palpable mass [5]. However, in many individuals-particularly those with obesity-a visible bulge may be absent, contributing to delayed diagnosis. In our reported case, the patient experienced symptoms for five years before receiving a definitive diagnosis, underscoring the need for heightened clinical suspicion when evaluating persistent or unexplained lower quadrant abdominal pain [6].

**Figure 4:**
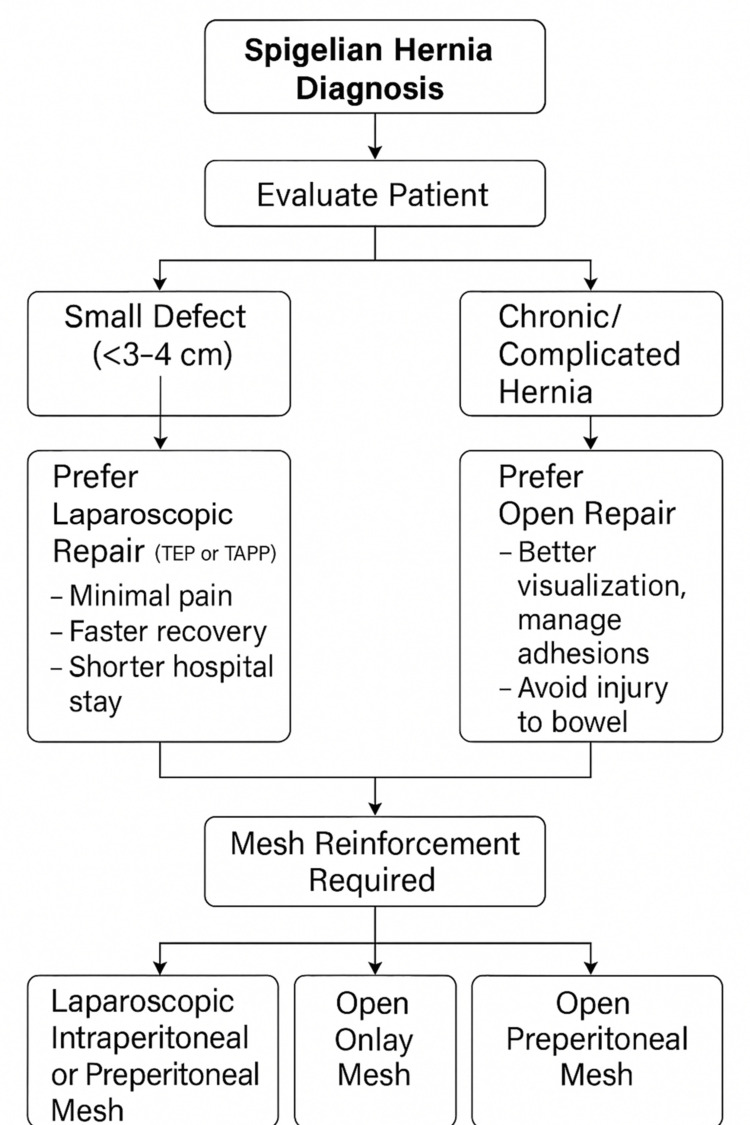
Flowchart outlining the management approach for Spigelian hernia. Patient evaluation guides the choice between laparoscopic or open repair based on hernia size and complexity. Mesh reinforcement, either intraperitoneal, preperitoneal, or onlay, is essential to prevent recurrence. TEP: totally extraperitoneal; TAPP: transabdominal preperitoneal

The flowchart outlining the choice of surgery for Spigelian hernia is shown in Figure 4.

Imaging plays a crucial role in identifying Spigelian hernias. While ultrasound can be helpful, especially in slender patients, its sensitivity varies depending on the operator. CT is the diagnostic modality of choice, offering precise visualization of the fascial defect and any herniated contents, as was evident in our case. Early imaging is essential not only for accurate diagnosis but also for planning surgical management [7].

Due to the narrow neck of the hernial sac, Spigelian hernias carry a higher risk of incarceration and strangulation, making surgical repair the preferred treatment. Both open and laparoscopic approaches are viable, with the selection depending on defect size, the surgeon’s skill set, and patient-specific considerations. In our case, an open mesh repair was performed successfully, offering the advantage of direct access to the defect and secure mesh fixation [8,9].

The patient had a smooth postoperative recovery and remained free of symptoms during follow-up, demonstrating the benefits of timely surgical management in avoiding complications and improving overall quality of life [10].

Our case is consistent with patterns reported in the literature, where Spigelian hernias often present with vague, chronic abdominal symptoms, leading to delays in diagnosis [11]. Imaging, particularly CT, remains the cornerstone for accurate preoperative identification. Although laparoscopic repair is increasingly favored for smaller defects, we opted for open mesh repair due to the chronic nature of the hernia, anticipated adhesions, and the patient's obesity, which could complicate laparoscopic access and increase operative risks-factors that have similarly influenced surgical decision-making in other reported cases [12,13].

In conclusion, this case underscores the importance of maintaining a high index of suspicion for Spigelian hernias in patients presenting with unexplained lower abdominal pain, particularly in those with risk factors like obesity or prolonged physical strain. Early diagnosis through appropriate imaging and timely surgical intervention are critical in preventing complications and ensuring favorable clinical outcomes.

## Conclusions

This case underscores the diagnostic challenges of Spigelian hernia, particularly when typical clinical signs are absent. What distinguishes this case is the prolonged, nonspecific symptomatology and the diagnostic delay despite multiple consultations, highlighting the importance of maintaining a broad differential in patients with persistent lower abdominal pain. Timely use of CT imaging was crucial in identifying the hernia and guiding successful laparoscopic repair. Clinicians and radiologists should remain vigilant for this rare entity, especially in patients with subtle or atypical presentations, to prevent avoidable complications through early intervention.
